# Hostile Interactions of Punjab Urial (*Ovis vignei punjabiensis*) towards Indian Gazelle (*Gazella bennettii*) during Feeding Sessions in Captive Breeding Settings

**DOI:** 10.3390/ani11051274

**Published:** 2021-04-28

**Authors:** Romaan Hayat Khattak, Liwei Teng, Tahir Mehmood, Ejaz Ur Rehman, Zhirong Zhang, Zhensheng Liu

**Affiliations:** 1 College of Wildlife and Protected Areas, Northeast Forestry University, Harbin 150040, China; romaanhayatkhattak@nefu.edu.cn (R.H.K.); zhang_zr@126.com (Z.Z.); 2 School of Natural Sciences (SNS), National University of Science and Technology (NUST), Islamabad 44000, Pakistan; tahime@gmail.com; 3 Snow Leopard Trust, Islamabad 44000, Pakistan; ejazrehman53@gmail.com

**Keywords:** captive ungulates, behavioural intolerance, social enrichment, mixed herds, *Ovis vignei punjabiensis*, *Gazella bennettii*, interspecific interactions, Pakistan

## Abstract

**Simple Summary:**

Multiple factors, both natural and anthropogenic, are driving most of the wild species to the verge of extinction across the globe. In order to conserve these threatened species, various conservation interventions and strategies are adopted, among which is re-introduction of captive stocks of species into the wild habitats where they vanished from. Captive breeding is one of the promising tools for endangered species preservation. Providing social enrichment to the captive stocks is an important step in the management, in particular for stocks, which are aimed for re-introduction. The subject species of this study, i.e., Punjab urial (*Ovis vignei punjabiensis*) and Indian gazelle (*Gazella bennettii*) are being reared in captivity with the aim of re-introduction. As there is scarcity of information regarding the behavioral aspects of captive species, especially ungulates, this study aimed at understanding the dynamics of their interactions. The current study reported that Punjab urial, being the dominant species, exerts itself on the submissive and subordinate species, the Indian gazelle. This negative interference can possibly lead to negative ramifications in the form of stress and injuries in the short term, while negative effects on population growth in the long term. Thus, this study recommends separate rearing of these species to eliminate the hazardous competition between them.

**Abstract:**

Natural wildlife habitats are regularly subjected to anthropogenic pressures for different purposes, which are heading the biodiversity towards drastic decline. Several endangered wild species are raised in captivity with the aim of re-introduction. In some instances, mixed herds’ rearing approach in captivity is adopted for providing social enrichment to captive stocks; however, the impacts of species on each other are least documented. We tested our prediction that keeping mixed herds of captive wild sheep and antelopes provides adequate social enrichment to the captive stocks: if interspecific interactions are balanced. In the current study, we studied the interspecific competition between mixed herds of captive Punjab urial (*Ovis vignei punjabiensis*) and Indian gazelle (*Gazella bennettii*) at Manglot Wildlife Park, Nowshera District, Khyber Pakhtunkhwa Province, Pakistan. We documented the negative effects of behavioural interference by Punjab urial on the feeding behaviour of Indian gazelle. The outcome of the current study revealed that Punjab urial are highly intolerant towards Indian gazelle, with high interference during feeding. Out of the total aggressive events, 77% (*N* = 1259) of events ended up with win/loss, in which Punjab urial dominated the Indian gazelle 3.5 times. Moreover, lopsided dominance by Punjab urial resulted in increased intraspecific competition among Indian gazelle (*p* < 0.001). Current study divulged Indian gazelle to be the subordinate species, with less intake of food. Instead of providing social enrichment by heterospecifics, the Punjab urial is negatively affecting the Indian gazelle, therefore, the results of our study discourage the practice of admix captive breeding for wild sheep and antelopes.

## 1. Introduction

Investigating the interspecific competitions among individuals is of utmost importance both in the wild and in captivity. These interactions for resources can affect the species’ growth rates, survival, and fitness [1]. Competition between species may be “exploitative competition” comprised of indirect negative interactions, resulting from common resources [2]. On the other hand “interspecific interference competition” is where one species limits and reduces other species’ ability to utilise the shared resources by its presence or agonistic interactions, and are ubiquitous [3]. Such interactions have been documented in a wide range of taxa, ranging from small mammals (rodents and bats) up to big mammals including carnivores and large herbivores [4,5,6,7,8,9,10]. Such agonistic interactions can possibly result in severe injuries and ultimately deaths [11,12], primarily when the competing species are restricted to confined areas. In such cases, the interspecific competitions lead to reduced intake of energy resulting in poor health, poor reproduction, and poor population growth [13]. Interspecific interactions are frequently lopsided, where the smaller species are more prone to lose the agonistic contests for resources with larger species, which are usually characterised by the physical attributes, i.e., strength and weapons (tusks, antlers and horns) [14]. In such cases, the smaller submissive species are expected to undergo behavioural changes to avoid dominant species [15]. However, such avoidance behaviours stop the smaller species from utilising the valuable food resources [15], and can have very unpleasant effects in captive environments, where the food is supplied in due quantity and time.

Overall, the interspecific competitions are believed to play a vital role in shaping the wild animal communities including ungulates [16,17]. In the wild natural environments, different ungulate species have been sympatric through evolution, which allows them to co-exist with one another [18]. In the natural wild habitats, the exploitative competition among ungulates is lessened, and sympatry is maintained by mechanisms like resource partitioning, consuming different forage species, or foraging other parts of the same species [19]. However, in captivity, resource partitioning is often influenced by behaviour, and thus, sympatric ungulates develop strong unpleasant aggressions to limit the food intake by other counterparts [19]. Interspecific aggression is much more common in carnivores [20], and least documented in herbivores [21,22].

The competitive success in animals is best defined as to have increased access to quality food [23,24], and controlling optimal shelter [25], which is mostly very true for wild animals in captivity with limited resources. Competitive failures may reduce the fitness of the loser animal, as it may be underprivileged by reduced access to food, dispossession of optimal shelter or receiving direct injuries from the powerful individual during the fight [26,27]. Thus, the losers and deprived individuals are much more prone to stress that can efficiently reduce the physiological processes, reproduction and survival probabilities and population growth rates [28,29]. 

During the last decades, populations of many wild species have been drastically declined and are facing increased extinction risks, and many of them are reared in captivity with the aim of re-introduction. This is also true for many wild animal species in Pakistan. Punjab urial (*Ovis vignei punjabiensis*) is a sub-species of Urial (*Ovis vignei)* and is exclusively endemic to Pakistan, while Indian gazelle (*Gazella bennettii),* a representative of antelopes; both are categorised endangered through local assessment, and are raised in captivity with the aim of re-introduction into the wild in Khyber Pakhtunkhwa (KP) Province of Pakistan [30,31,32]. Even though the institutions keeping wild animals in captivity try their best to provide adequate food, it is challenging to balance overfeeding and reduce harmful effects of competitions [29]. Therefore, competition in captivity is much higher than in the wild, with higher densities and lower food availability [33]. Extensive competitions arise in captivity when loser animals fail to secure sufficient food and potentially suffer from unpredictable supply [34,35,36], while the dominant animals overfeed. With the increase of competition for food, aggression levels rise and subsequently, the stress [36]. For promising captive breeding programmes, the goal is to provide enough food for each animal and maintain an acceptable level of competition [1]. To reduce the competition among conspecifics, an alternative option is to keep animals solitary, however, lack of social interactions will have adverse effects on stocks which are supposed to be used in re-introduction programmes [37,38].

In captive breeding practices, along with the provisions of appropriate environments that focus on physical aspects (enclosure size and design) and food enrichment (variety, processing and presentations) [39], social enrichment is also potentially significant [40]. Usually, the social enrichment is provided by the conspecifics, however, the other species (heterospecifics) may also have the potential for boosting animal welfare [40]. Keeping wild animals in mixed species groups is one such way to provide animals with complex physical and social environments [39], and an acceptable level of competitions where there is a balance between two species; these are signs of optimal animal welfare [41]. However, extreme interspecific competitions will result in many unpleasant scenarios like aggressions and stress [41], and may have adverse effects on the subordinate species and thus reflect poor management. We assumed keeping mixed herds of captive wild sheep and antelopes provides adequate social enrichment to the captive stocks: if interspecific interactions are balanced. The current study was thus designed to investigate (1) the interspecific interactions during feeding between mixed herds of captive breeding Punjab urial and Indian gazelle, and (2) to find the dominance and compatibility between these two species.

## 2. Materials and Methods

### 2.1. Study Area 

The current study was conducted at the captive breeding facility located at Manglot Wildlife Park (MWP) (33°45′19″ N, 72°0′15″ E), Nowshera district, KP province Pakistan. The study area has a predominant scrub forest with semi-arid climate. The average annual temperature recorded is 24.4 °C with an average rainfall of 532 mm. Mixed herds of Punjab urial and Indian gazelle are raised in an enclosure encompassing an area of 15,793 m^2^. In addition to the two species mentioned above, three males of Mouflon sheep (*Ovis orientalis*) were also present in the same enclosure. However, before conducting this study, the three male Mouflon sheep were shifted by the wildlife and park authorities into a zoo located in the Peshawar district of KP province. This study was carried out from 26 November–26 December 2020. 

### 2.2. Study Animals

During the study period, a total of 15 individuals were present in the enclosure, including 8 Punjab urial and 7 Indian gazelle. It was not allowed to restrain or capture the study animals for tagging. Therefore, we performed a preliminary survey to identify every individual based on the demographic and physical features (Table 1). Codes were allotted to each particular individual.

### 2.3. Control Group

We recognised that this study lacks a control group for direct comparison, as there were no opportunities to separate the animals into single-species enclosures for the purposes of this research. An alternative would be to consider a wild population as a control group because urial and gazelle rarely occupy the same spaces in the wild. However, we could not use the wild case as a control group because although urial and gazelle exist independently in the wild, i.e., equivalent to single-species enclosures and can be considered a suitable control group; yet, they occur at such low densities that it was not realistic to locate and assess wild individuals, and we also expect captive and wild individuals to have some behavioural dissimilarities.

### 2.4. Behavioural Observations

During this study, all the data were collected only during feeding hours. Study animals were fed twice a day (morning and late afternoon), and all the threat displays were recorded on specified sheets. While recording the events, it was assumed that when a receiver left the feed due to the actor’s threat, it was considered a “displacement event”. It did not matter if the receiver left the feed for a very short time. If a displacement event occurs, that was considered a “win” for actor and a “loss” for the receiver. On the other hand, if the receiver did not leave the feed in response to the actor’s threat, it was considered an “event with no outcome”. A single vantage point outside the enclosure was selected for observations. The vantage point was converted into a temporary camouflaged hide to minimise observers’ effect on animals’ behaviour. Moreover, data was recorded until it was assured that all animals left the feed and were no more interested in eating. Start and end time of each particular observation was recorded.

On the first day of observation, the feed was put down in two points as per regular practice by the keepers. However, due to animals’ clumping, it was difficult to identify the actor and receiver animal quickly and to adequately record the events. Therefore, in the following days, the number of feed points were increased up to 5, at a distance of approximately 15 m apart.

### 2.5. Statistical Analysis

All the data were analysed by using matrices [42] and descriptive statistics. To investigate the effects of Punjab urial on Indian gazelle, we pooled out the interspecies interactions events data. To find out the difference between the interspecific and intraspecific aggressions and sexes, we performed the non-parametric McNemar’s Chi-square test, by using function mcnemar.test in R-package version 3.5.1. Significance level was set at *p* < 0.05.

## 3. Results

### 3.1. Overall Interactions 

With an effort of 23.16 h, a total of seven threat behaviours (Table 2) were recorded, yielding a total of 1635 agonistic interaction events among all the individuals. Out of the total events, 77% (*N* = 1259) of events ended up with win/loss, and 23% (*N* = 376) of events were ended with no outcome (Table 3). Out of the total events ended up with wins/loses, Punjab urial won 67.8% (*N* = 854), and Indian gazelle won 32.2% (*N* = 405) events. Among Punjab urial, UM3 scored the highest number of wins with 36.12% (*N* = 309), followed by UM1, UM2 and UF2, with a win percentage of 24.35% (*N* = 208), 12.29% (*N* = 105), and 11.47% (*N* = 98), respectively. 

Among Indian gazelle, GM1 scored the highest win percentage of 37.77% (*N* = 153) followed by GM2 and GF3, with 29.38% (*N* = 119) and 16.04% (*N* = 65), respectively.

### 3.2. Urial–Gazelle Interactions

**Figure 1:**
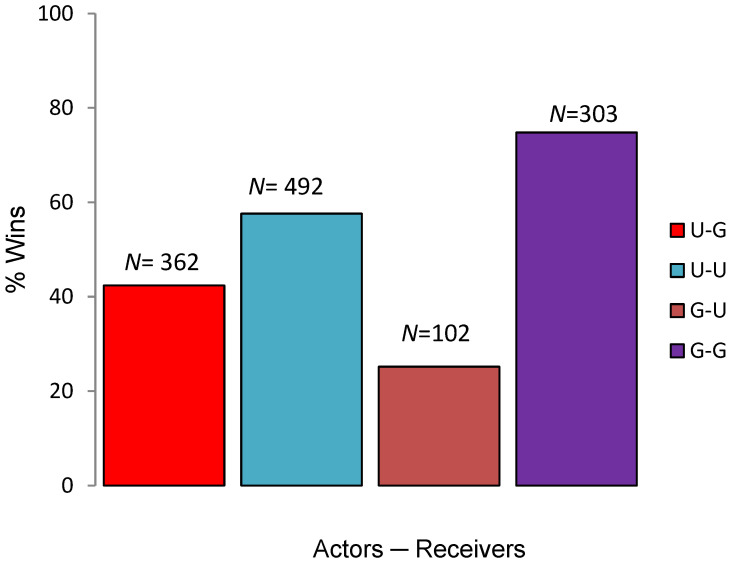
Percentage wins resulted from encounters between Urial–Gazelle, Urial–Urial, Gazelle–Urial and Gazelle–Gazelle (total count on the top of bar).

Out of total wins (*N* = 854) by Punjab urial, 42.38% (*N* = 362) were attained against Indian gazelle (U–G). Out of total wins by Indian gazelle, reversal wins (G–U) against Punjab urial were 25.18% (*N* = 102) (Figure 1). Results obtained from McNemar’s Chi-square test revealed that there was significant difference between intraspecific and interspecific aggression levels (χ^2^ = 145.38, df = 1, *p* < 0.001).

**Figure 2:**
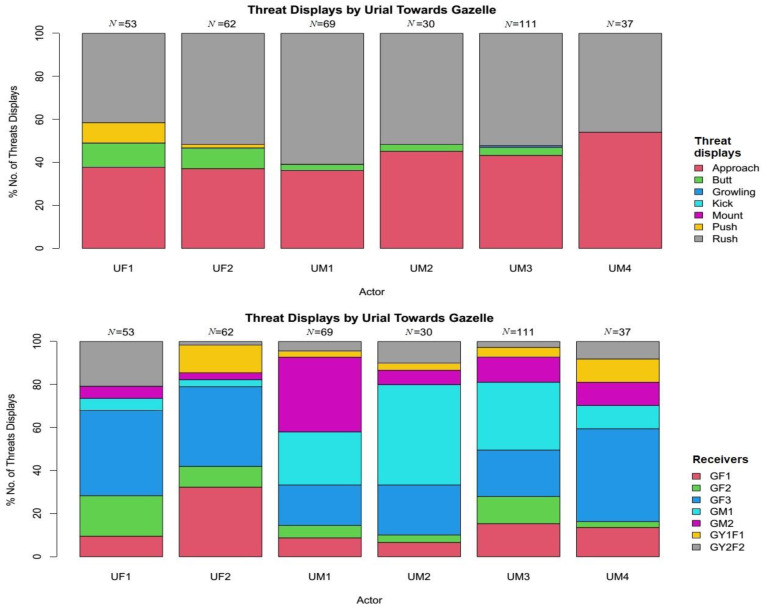
Threat displays of Punjab urial towards Indian gazelle with respect to threats variety and receivers (behaviours with 0% included as a reference to overall threats).

**Figure 3:**
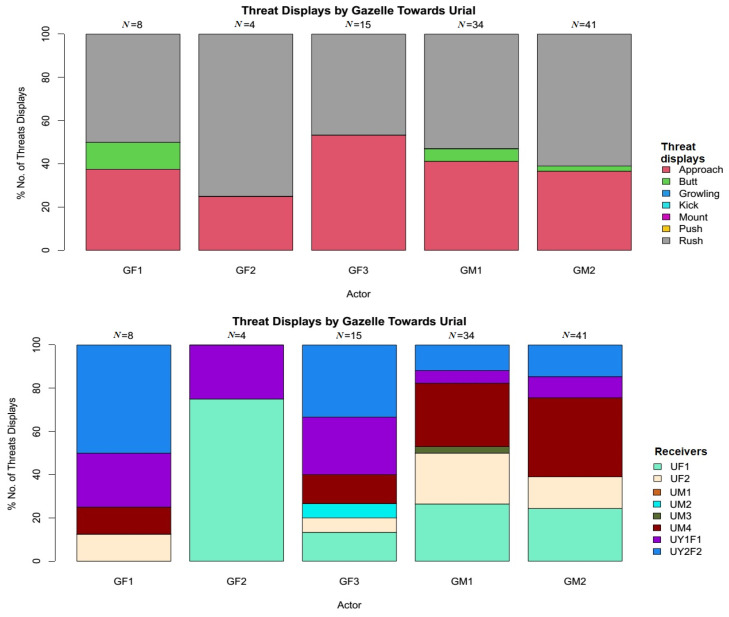
Threat displays of Indian gazelle towards Punjab urial with respect to threats variety and receivers (behaviours with 0% included as a reference to overall threats).

There was a significant difference among all the displayed threats behaviours, showing significant preference to rush behaviour by both species (χ^2^ = 227.64, df = 77, *p* < 0.001). Variety of threats displayed by Punjab urial towards Indian gazelle was diverse, including rush 51.08% (*N* = 188), approach 41.3% (*N* = 152), butt 5.43% (*N* = 20), push 1.90% (*N* = 7), and growling 0.27% (*N* = 1) (Figure 2). However, Indian gazelle displayed only rush 56.31% (*N* = 58), approach 38.43% (*N* = 40), and butt 4.85% (*N* = 5) (Figure 3). 

## 4. Discussion

### 4.1. Overall Interactions

The current study results reveal that both types of competitions, i.e., exploitative and interference competition, simultaneously exist between captive breeding herds of Punjab urial and Indian gazelle. Same food resources for both species seem to be the main reason for both types of competitions between the species. It has been observed that exploitative and interference competition between the competing species quickly alternates if the resources are limited [43]. In the current study, interspecific interactions seem lopsided, ranking Punjab urial as a dominant species and Indian gazelle as a subordinate species. The main reasons for such lopsided competitions are the size, strength, and weapons possessed by the dominant species [14]. This statement favours Punjab urial, rather than Indian gazelle. 

Both species displayed similar threat displays, except kick and growling shown by Indian gazelle and Punjab urial, respectively. Only urial males displayed growling, which is commonly reported as a threat display in carnivores [44], and less prominent in ungulates. However, growling has been confirmed as agonistic display in Punjab urial rams [45], and other sheep species [46]. The findings in the studies mentioned above validate our results. Both Punjab urial and Indian gazelle performed the “rush” behaviour as the most powerful and determined threat displays (χ^2^ = 227.64, df = 77, *p* < 0.001) adding the highest percentage to their wins. Punjab urial displayed a total of 332 rush displays, contributing 25.97% (*N* = 327) to their overall win.

On the other hand, Indian gazelle displayed “butt” with the highest frequency (*N* = 189) with a winning ratio of 8.89% (*N* = 112). However, the win ratio attributed to “rush” (*N* = 174) was higher (12.86%, *N* = 162) than “butt”. We assume that in both species that high rush behaviours were to establish dominance on the submissive individuals, which is true for gregarious wild artiodactyls [47]. Moreover “rush” subsequently increased the “butting” among subordinate individuals by restricting them to the single feed point. It has been reported that frequent and sometimes lethal butting occurs among bovids when they are clumped on limited feed at ground [47]. Punjab urial, especially rams, showed the highest number of aggressive “approaches” (*N* = 285), adding 19.38% (*N* = 244) to their overall wins. We assume that the large body size of Punjab urial was the main factor to establish dominance over the conspecifics and Indian gazelle. Thus, frequently moving here and there to keep a hold on multiple feeding sites resulted in lopsided interference competition [14]. Out of total “approaches” made by Punjab urial, most (*N* = 151) were directed towards Indian gazelle, with 3.75 times fewer reversal approaches (*N* = 40) from Indian gazelles towards Punjab urial. These results indicate the subordinate rank [48], for Indian gazelle concerning Punjab urial. Surprisingly every “approach” by either species towards each other resulted in a win for the respective actor and a loss for the receiver.

The present study showed that many agonistic interactions during feeding resulted in displacements. However, most of these were resolved by the recipient’s submissive behaviors without any overt threat response, especially by Indian gazelle. Similar findings have been reported for other wild ungulates species [49,50,51]. In both species, males were highly aggressive. In Punjab urial, the highest threat was displayed by UM3 (*N* = 389) followed by UM1 (*N* = 260) and UM2 (*N* = 141). UM3, being a sub-adult male in the study, was highly aggressive, probably because it was more alert and established dominance among the older males avoiding any possible threat from the younger individuals [52]. During the study period, UM1 and UM2 age were 6 years and 5 years, respectively; however, UM3 was 3.5 years old. It has been found that in wild sheep and goat, the ability to win feeding contests in males declines after 5 years of age [48]. In the case of Indian gazelle GM1 and GM2, both adult males were highly aggressive. The dominance rank in antelopes is related to age, body size, and horns. Usually, the adult males are dominant over the other conspecifics [53,54,55,56].

### 4.2. Urial–Gazelle Interactions 

The current study showed that Punjab urial is highly intolerant towards Indian gazelle. Indian gazelle is a nomadic and semi-nomadic species of deserts, sand dunes, semi-deserts, and arid range lands, adapted to movements searching for ephemeral and seasonal forage [57]. However, Punjab urial is resident to its habitats with comparatively higher elevations, and forages on all kinds of vegetation ranging from grasses to shrubs and trees [58]. We assume that based on habitat and diet preferences, both these species’ ecological niche is a bit different. Species with different ecological niches can easily co-exist [59,60], but that is true when living in their natural wild habitats.

Moreover, according to the “Optimal foraging theory” [61,62], both these species have narrowed their niches and are more concentrated on the same food source. Thus, interspecific encounters are increased manyfold [63]. Based on the results, we assume a constant overlap due to the same feed sources in captivity exists between Punjab urial and Indian gazelle. This existing overlap amplifies the potential for interspecific competition between the two species [17,64,65,66], making Punjab urial as the more intolerant species due to its bigger size and high energy demands.

Among the horned ungulates, rush and approach towards the opponent are the powerful and preferred threat displays [67,68] to conquer food or mates. The dominant individuals predominantly perform these two threat displays and anticipate the actors’ wins [67]. The results obtained in the current study are in agreement with the above statements. Both species preferred “rush” and “approach” against each other (Figure 2 and Figure 3). However, Punjab urial aggression is many folds higher, which demonstrates its dominance over Indian gazelle. Gazelles, in particular, when approaching or rushing at opponents, utter and make relatively louder and repeated snoring [67]. However, in the current study, no such behaviour was recorded for gazelles; instead, Punjab urial performed this threat display both towards conspecifics and Indian gazelle.

In both groups, the highly displaced individuals were females (χ^2^ = 413.8, df = 1, *p* < 0.001) (Figure 2 and Figure 3). Similar results for the high displacement of females from feeding sites have been reported for other ungulates [63]. Usually, the females need to consume more and have a high energy food intake to satisfy high energy demands due to gestation and lactation [69]. In the current study, except one female (GF3), all the other urial and gazelle females were with their lambs and fawns. Therefore, we assume that females in both herds made much movement to access more food, and subsequently faced higher encounters and displacements. Among Punjab urial males, a yearling male (UM4) was the only male individual highly displaced (*N* = 28) by Indian gazelle. The UM4 was highly displaced by the dominant conspecifics, leading to higher approaches to other feeding sites; thus, subsequently becoming an easy target for the adult and more experienced gazelles. 

According to the experiences and reports published by the American Zoo Association (AZA) regarding mixed species exhibits, these reveal that, in so many cases, the mixed species exhibits was not a successful practice. Particularly, in most of mixed species exhibits, *Gazelle* species were separated from other ungulates, either due to exerting extreme levels of aggression or receiving highly lopsided aggression from heterospecifics. Similar evidences were also reported for Mouflon sheep which is a close relative to Urial, against other ungulates (http://www.azaungulates.org/mixed-species, accessed on 8 March 2020).

## 5. Conclusions

Our study indicates that Punjab urial stands out dominant over Indian gazelle owing to its physical size and strength. We assume such high suppression rates for Indian gazelle by Punjab urial can potentially affect the feeding behaviour, growth rates, and later reproduction. Although keeping mixed herds in captivity increases social enrichment and animal welfare, only the different species maintain positive or balanced offensive interactions. Based on the results obtained in the current study, we assume that, in captivity, Punjab urial is not compatible with Indian gazelle, and such high levels of aggression can possibly result in injuries or even death of the weak counterparts. As both the species are locally endangered and are raised with the aim of re-introduction; therefore, to avoid any damage or loss to the existing stocks, our findings discourage practising combined captive breeding of wild sheep and antelopes. Social enrichment, in this particular case, seems better if provided through conspecifics. Furthermore, it is strongly recommended to also provide these animals with other environmental enrichments such as physical, sensory and dietary.

## Figures and Tables

**Table 1:** Study animal’s description with respect to demography and allotted id’s.

s.no	Animal ID	Demography	Description
1	UM1	Adult urial male	Horns curled downward, dark reddish-brown coat color, conspicuous vertical white saddle mark, long extended ruff comprised of mixed white and black hairs
2	UM2	Adult urial male	Horns curled downwards, reddish coat color, saddle mark absent, extensive ruff with mostly white hairs in the throat and black hairs on the chest area
3	UM3	Sub-adult urial male	Horns comparatively shorter and curled downward, saddle mark absent, ruff was like a flat black strip extended through the chest without extensively long hairs
4	UM4	Yearling urial male	Sickle-shaped horns curled backward, somewhat greyish coat color, ruff not conspicuous.
5	UF1	Adult urial female	Grey coat color, one horn broken, with young UY1F1
6	UF2	Adult urial female	Grey coat color, both horns present, with young UY2F2
7	UY1F1	Urial lamb	Offspring of UF1, comparatively healthy and bigger than UY2F2
8	UY2F2	Urial lamb	Offspring of UF2, comparatively weaker and smaller than UY1F1
Indian gazelle			
1	GM1	Adult gazelle male	Dark greyish sandy color, longhorns
2	GM2	Adult gazelle male	Greyish sandy colour, horns were comparatively shorter than GM1
3	GF1	Adult gazelle female	Greyish sandy colour, with young (GY1F1)
4	GF2	Adult gazelle female	Greyish sandy colour, with young (GY2F2)
5	GF3	Adult gazelle female	Greyish sandy colour, without any young
6	GY1F1	Gazelle fawn	Offspring of GF1, comparatively bigger than GY2F2
7	GY2F2	Gazelle fawn	Offspring of GF2, comparatively smaller than GY1F1

**Table 2:** Recorded threat behaviours, displayed by Punjab urial and Indian gazelle during observations.

Behaviour	Description
Approach	The actor walks straight towards the receiver.
Butt	The actor bashed the receiver’s body in rump or sides.
Push	The actor pushes the receiver, either frontal head-to-head push or receiver attains a push from actor at any part of the body.
Growling	The actor turns its head towards the receiver and gently growls without butting.
Kick	The actor kicks the receiver with hind leg.
Mount	The actor mounts on the receiver.

**Table 3:** Number of aggressive interactions (wins/losses) among Urial–Gazelle, Urial–Urial and Gazelle–Gazelle (χ^2^ = 846.4, df = 165, *p* < 0.001).

	Loser															
Winner	GF1	GF2	GF3	GM1	GM2	GY1F1	GY2F2	UF1	UF2	UM1	UM2	UM3	UM4	UY1F1	UY2F2	Total Wins
GF1	0	0	22	3	3	0	6	0	1	0	0	0	1	2	4	42
GF2	1	0	11	5	0	4	1	3	0	0	0	0	0	1	0	26
GF3	6	6	0	1	2	19	16	2	1	0	1	0	2	4	5	65
GM1	7	12	34	0	25	15	26	9	8	0	0	1	10	2	4	153
GM2	13	4	24	5	0	24	8	10	6	0	0	0	15	4	6	119
UF1	5	10	21	3	3	0	11	0	4	0	0	0	11	0	9	77
UF2	20	6	23	2	2	8	1	11	0	0	1	0	12	12	0	98
UM1	6	4	13	17	24	2	3	8	17	0	9	37	37	13	18	208
UM2	2	1	7	14	2	1	3	13	6	7	0	11	24	9	4	105
UM3	17	14	24	35	13	5	3	35	25	27	19	0	56	19	17	309
UM4	5	1	16	4	4	4	3	4	0	0	0	0	0	9	7	57
Total Losses	82	58	195	90	78	82	81	95	68	34	30	49	168	75	74	1259

## Data Availability

All the data sets obtained are presented in this article.
